# Intraluminal oxygen can keep small bowel mucosa intact in a segmental ischemia model

**DOI:** 10.1038/s41598-024-64660-x

**Published:** 2024-06-14

**Authors:** Guido Trentadue, Peter B. F. Mensink, Claudius Kruse, Bernward Reszel, Gursah Kats-Ugurlu, Tjasso Blokzijl, Jan Willem Haveman, Klaas Nico Faber, Gerard Dijkstra, Uvo M. Hölscher, Jeroen J. Kolkman, Gisbert Knichwitz

**Affiliations:** 1 Department of Gastroenterology and Hepatology, University of Groningen, University Medical Center Groningen, Hanzeplein 1, 9713GZ Groningen, The Netherlands; 2 Department of Internal Medicine and Gastroenterology, Medisch Spectrum Twente, Enschede, The Netherlands; 3 Department of Anaesthesiology, University Hospital Muenster, Muenster, Germany; 4 Department of Pathology, University of Groningen, University Medical Center Groningen, Groningen, The Netherlands; 5 Department of Laboratory Medicine, University of Groningen, University Medical Center Groningen, Groningen, The Netherlands; 6 Department of Surgery, University of Groningen, University Medical Center Groningen, Groningen, The Netherlands; 7 Münster University of Applied Sciences, Steinfurt, Germany; 8 Present Address: Department of Anaesthesiology and Operative Intensive Medicine, Franziskus Hospital, Intensive Care Medicine, Bielefeld, Germany; 9 Present Address: CERES GmbH, Clinical Evaluation and Research, Lörrach, Germany; 10 Present Address: Dreifaltigkeits-Krankenhaus Cologne, Klinik Für Anästhesiologie, Intensivmedizin Und Schmerztherapie, Cologne, Germany; 11 Berufliche Fortbildungszentren der Bayerischen Wirtschaft (bfz) gGmbH, München, Germany

**Keywords:** Small intestine, Animal disease models, Ischemia, Intestinal Mucosa, Oxygen, Preservation, Intestinal transplantation, Gastrointestinal models, Intestinal diseases, Experimental models of disease, Translational research

## Abstract

Intestinal preservation for transplantation is accompanied by hypoperfusion with long periods of ischemia with total blood cessation and absolute withdrawal of oxygen leading to structural damage. The application of intraluminal oxygen has been successfully tested in small-animal series during storage and transport of the organ but have been so far clinically unrelatable. In this study, we tested whether a simple and clinically approachable method of intraluminal oxygen application could prevent ischemic damage in a large animal model, during warm ischemia time. We utilised a local no-flow ischemia model of the small intestine in pigs. A low-flow and high-pressure intraluminal oxygen deliverance system was applied in 6 pigs and 6 pigs served as a control group. Mucosal histopathology, hypoxia and barrier markers were evaluated after two hours of no-flow conditions, in both treatment and sham groups, and in healthy tissue. Macro- and microscopically, the luminal oxygen delivered treatment group showed preserved small bowel’s appearance, viability, and mucosal integrity. A gradual deterioration of histopathology and barrier markers and increase in hypoxia-inducible factor 1-α expression towards the sites most distant from the oxygen application was observed. Intraluminal low-flow, high oxygen delivery can preserve the intestinal mucosa during total ischemia of the small intestine. This finding can be incorporated in methods to overcome small bowel ischemia and improve intestinal preservation for transplantation.

## Introduction

Of the abdominal organs, the intestine is the most sensitive for ischemia due to the high oxygen consumption of its mucosa, and the most immunogenic due to its intrinsic contamination and large amount of immune-cell population in the body^1^. Ischemia of the bowel will eventually drive multiple organ failure^2^.

Organ preservation in intestinal transplantation (ITx) is unavoidable and leads to ischemia reperfusion injury which eventually results in worse graft survival. During the donor procedure there is a slow decrease in the temperature of the organs starting with the vascular flush of cold preservation solution^3^. The cold-ischemia time (CIT), which begins at this point, consists of the period of cold storage and graft inspection before implantation. A period of warm-ischemia time (WIT) exists before restoration of the blood flow in the recipient and lasts an average of 47 (± 15) minutes in our Dutch centre, and an average of 45 (± 11) in several reports from the literature^4–7^.

Ischemia generates cell swelling, adenosine triphosphate depletion and accumulation of reactive oxygen species^8^. Preservation injury and ischemia–reperfusion injury (IRI) are the results of the damage that the (intestinal) graft suffers during and after transplantation^9^. The destruction of the epithelial lining serves as passage for commensal bacteria to translocate to the lamina propria. These occurrences will eventually determine the early and late outcomes of the treatment^9–11^. The ischemic state produced by WIT is unavoidable, and its effects are as grave, if not worse, than those of CIT, mainly because of the metabolic rates occurring at normothermia^4,12^.

Standard transplant procedures and current research do not focus either on improvement of conditions during WIT, or on the most effective way to reach the intestine: its lumen. Furthermore, the available research models differ from clinical scenarios, so the prospects of clinical translation are low. An approach to solve some of these problems is to preserve the intestine during ischemia with luminal treatments. This is particularly advantageous in ITx, when the organ is easily accessible, and ischemia is a programmed occurrence. In this study, we modified a large-animal model of local no-flow ischemia in a small intestine segment of clinically relevant length and size and used a low-flow and high-pressure intraluminal oxygen delivery system. The goal of the study was to address three major points with these experiments: (1) overcoming temporary warm ischemia and the consequent mucosal damage by (2) intraluminal oxygenation in (3) a large animal model that could be clinically translatable by use of appropriately-sized tools for human bowels.

## Materials and methods

Animal experiments were performed in the University of Muenster in compliance with the institutional review board for the care of animals, in accordance with the National Institutes of Health guidelines for ethical animal research. Data acquisition from the gathered samples was performed both in the University of Muenster and the University Medical Center Groningen. There are additional materials and methods described in the Supplementary Information section including gene expression studies and further analyses on immunohistochemistry. The study is reported in accordance with ARRIVE guidelines.

### Animal preparation

The model herewith described was adapted from an existent, successful, large pig model of ischemia previously described by this group^13–15^. Twelve 1-year-old female domestic pigs were fed on a standard diet and fasted overnight and randomised into the different groups. Premedication consisted of intramuscular 5 mg/kg ketamine, 3 mg/kg azaperone and 0.02 mg/kg atropine. Anaesthesia was induced using thiopentone 5–6 mg/kg, administered through an ear vein. The pigs were intubated and ventilated using the closed system ventilator Physioflex (Draeger, Lübeck, Germany) with 50% oxygen in air and a tidal volume of 10 mL/kg. The respiratory rate was set and, if required, adjusted to maintain normocarbia. Anaesthesia was maintained throughout the experiment applying 1.8 ± 0.07% isoflurane. An arterial catheter was inserted into the right carotid artery, and a thermodilution pulmonary artery catheter was inserted via the right jugular vein.

**Figure 1:**
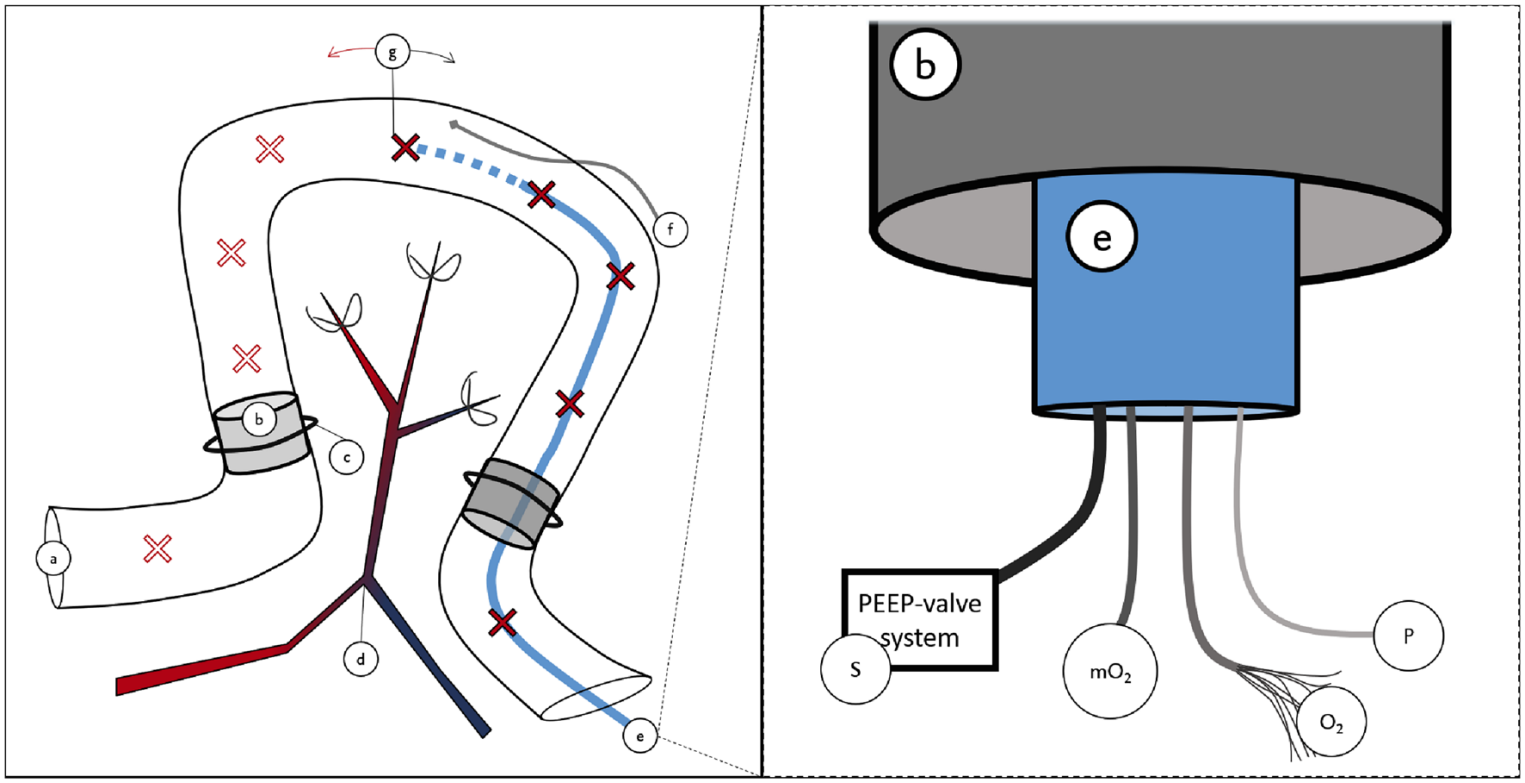
Schematic drawing of the small bowel segmental ischemia model. Left: (a) Small bowel lumen—proximal end; (b) intraluminal tubes; (c) ligatures; (d) mesenteric blood vessels, ligated at distal branches; (e) intraluminal catheter (oxygen delivery at the tip)—distal end; (f) fibre-optic sensor for O_2_, PCO_2_ and pH measurements; (g) histology sampling points, in centimetres: centre (0), − 10 and + 10 cm, − 20 and + 20 cm, − 30 and + 30 cm, and measurements outside the ischemic area, − 60 and + 60 cm. Right, zoomed view of the (distal) end to show catheters: (b) plastic tube to allow free passage of luminal gases; (e) O_2_-supplying tube, containing an infusion catheter (S) connected to a positive end-expiratory pressure (PEEP)-valve system, an O_2_-measurement catheter (mO_2_), the oxygen supply tube with inner catheters (O_2_), and a pressure-sensor catheter (P).

A midline laparotomy was performed, and 100 cm of the small bowel were chosen, starting 50 cm behind the ligament of Treitz. From this section, a segment of 70 cm was selected, and opened on both ends to place plastic tubes with a length of 2 cm and a diameter of 1 cm in the lumen on either side to allow free passage of luminal gases during outer-mural ligation of wall vessels (Fig. 1). These mesenteric ligatures were placed around the tubes to block the intramural collateral network. Four catheters were introduced though the proximal tube with their tips at the middle of the experimental segment (“centre” from here on). The proximal opening was closed after insertion of the catheters. The mesenteric vessels were also ligated at 10 cm intervals. Complete cessation of blood flow of the selected bowel segment could be achieved with closure of these ligatures.

### Monitoring materials

The catheters placed in the lumen were an oxygen supply tube with 9 separate inner catheters to ensure permanent low volume, high pressure flow and avoid clogging, no balloon at the end, connected to the central oxygen supply (Fig. 1, “O_2_” 8 + 1-lm silicone “manometry” catheter, 13 Fr; Medical Measurement Systems BV, Enschede, The Netherlands); an O_2_ measurement catheter (Fig. 1 “mO_2_” O2C catheter, 100 cm length, 13 Fr; LEA Medizintechnik GmbH, Giessen, Germany); a pressure sensor (Fig. 1 “P” 14 Fr; Becton Dickinson GmbH, Heidelberg, Germany); and a standard infusion catheter (Fig. 1 “S” Baxter Germany GmbH, Unterschleißheim, Germany) connected to a positive end-expiratory pressure (PEEP)-valve system which was set on 2 kPa, to avoid intraluminal overpressure. A fibreoptic sensor (Fig. 1 “f”) for PCO_2_ and pH measurement (Paratrend 7®; Biomedical Sensors, Wycombe, United Kingdom) was introduced near the centre of the experimental section.

### Treatment and sample and data collection

After 30 min, ischemia was induced by tightening the mesenteric ligatures (t = 0). In the treatment group (n = 6) oxygen therapy was started at t = 0. The oxygen therapy consisted of 100% oxygen delivered at low-flow (5 ml/min) and high-pressure (2 bar) for 120 min, more than doubling the average WIT of our transplant centre. The sham group (n = 6) did not receive treatment after the ligation. The abdomen was closed immediately after t = 0. During the experiment, the body temperature was maintained at 38 ± 1 °C. Heart rate, mean arterial pressure, temperature and intraluminal PCO_2_, PO_2_ and pH were measured continuously. The pulmonary capillary wedge pressure was measured every 30 min, simultaneously with blood gas analysis (pH, pCO_2_ and PO_2_) and haemoglobin concentration from arterial blood samples (ABL 520 blood gas analyser, Radiometer Copenhagen, Copenhagen, Denmark). Lactate was measured from peripheral venous blood. After 120 min (t = 120) the abdomen was re-opened. Samples for histological examination were taken from two areas at the centre of the small intestine segment, and 10, 20, and 30 cm, both proximal and distal, from that location. Samples were taken also outside the ischemic area, 100 cm proximal and distal from the centre, and considered control tissue (Fig. 1, left).

### Histology

The specimens were immediately fixed in 4% formaldehyde-buffered solution. They were embedded in paraffin, cut into 4 µm-thick slices, and stained with haematoxylin and eosin for morphometric analysis. One investigator, blinded, independently examined the specimens, using scanned, high-resolution files of at least two slides per sample (× 400; Hamamatsu C9600-12 slide scanner and NDP.scan 2 software for viewing and measuring; Hamamatsu Photonics, Japan). The morphological changes in the mucosa were categorised according to the 8-grade scale introduced by Chiu et al*.*^16,17^ and further developed by Park et al*.*^18^ (Chiu/Park score, see Supplementary Table S1). To account for the patchy nature of ischemic lesions, scores were taken from twenty randomly selected high-power fields (400x) per sample. Additionally, the percentage of healthy surface epithelium of the full sample present on each slide (Chiu/Park score ≥ 3) and, villous height, crypt depth and villous to crypt ratio (VCR) were calculated^19^.

### Immunohistochemistry

Glass slides with 4 µm-thick tissue samples were pre-treated for epitope retrieval (citrate, pH 6). Subsequently, samples were blocked and incubated with the primary antibody for one hour. The primary antibodies were anti-hypoxia-inducible factor-1-alpha (HIF-1α, 1:200 dilution, [mgc3] ab16066) and anti-villin-1 (1:100, ab52102) from Abcam (Cambridge, London, United Kingdom). Rabbit anti-mouse immunoglobulin and goat anti-rabbit immunoglobulin, both peroxidase-labelled (1:50, Dako, Agilent, Santa Clara, CA, USA), were each incubated for 30 min as secondary and tertiary antibodies. 3,3′-diaminobenzidine was used as substrate.

### Statistics

Data analysis was performed in blind fashion.

The results of the luminal gas measurements and systemic haemodynamic values, acid–base variables and biochemical markers are presented as mean ± SD. To find significant changes between measurements of different time points, paired student T tests were performed, (p < 0.05 was considered significant).

The distribution of the individual Chiu/Park scores for the control samples were tested for normality with the Kolmogorov–Smirnov and Shapiro-Wilks tests. To check for differences between all control segments, their individual scores underwent Kruskal–Wallis and Dunn’s tests. Similarly, to check for differences between pairs of central segments, a Mann–Whitney test was performed. Consequently, the mean scores of the 20 microscopic fields of each individual animal were used for statistical analyses thereafter. Results are presented as median and range (minimum–maximum), evaluated using a nested one-way ANOVA and Tukey´s multiple comparisons tests (p < 0.05 was considered significant). The villous height, crypt depth and VCR are presented as median and interquartile range (25–75%) and was evaluated with Kruskal–Wallis and corrected for multiple comparisons with Dunn’s test.

Data analyses and statistical tests were performed using Excel (Microsoft, Redmond, WA, United States), GraphPad Prism 8.0 (GraphPad Software, Inc., La Joya, CA, United States) respectively.

## Results

### Acid–base variables and biochemical markers between sham and treatment group were similar

No difference in pre-manipulation body weight between both groups was found (sham, 73.5 kg, range 72–79 and treatment, 74.5 kg, range 73–79). While the heart rate remained equal throughout the experiment, mean arterial pressure increased over time on the treated subjects (p = 0.006, Supplementary Figure S1). The arterial pH, pCO_2_, pO_2_, haemoglobin, glucose, Na^+^, and Cl^−^ did not differ between groups and remained stable. The values of Ca^2+^, HCO_3_ increased over time in the untreated group, as did arterial K^+^ levels in both groups (Supplementary Table S2).

### Intraluminal PO_2_ increased and PCO_2_ decreased in the treatment group

In the untreated group, the intraluminal PO_2_ dropped to nearly 0 mmHg within 30 min after ligation, and the intraluminal PCO_2_ peaked to 135 mmHg after 30 min. In the treatment group, the intraluminal PO_2_ raised from 126 mmHg directly after ligation, to a maximum of 679 mmHg after 90 min. The intraluminal PCO_2_ decreased gradually from 61 mmHg at the start, to 43 mmHg after 120 min. The intraluminal pressure during the experiments did not exceed 1 kPa. The luminal pH decreased to 6.6 after 60 min, comparable in both groups.

### Luminal oxygen preserved the small intestine macroscopically

**Figure 2:**
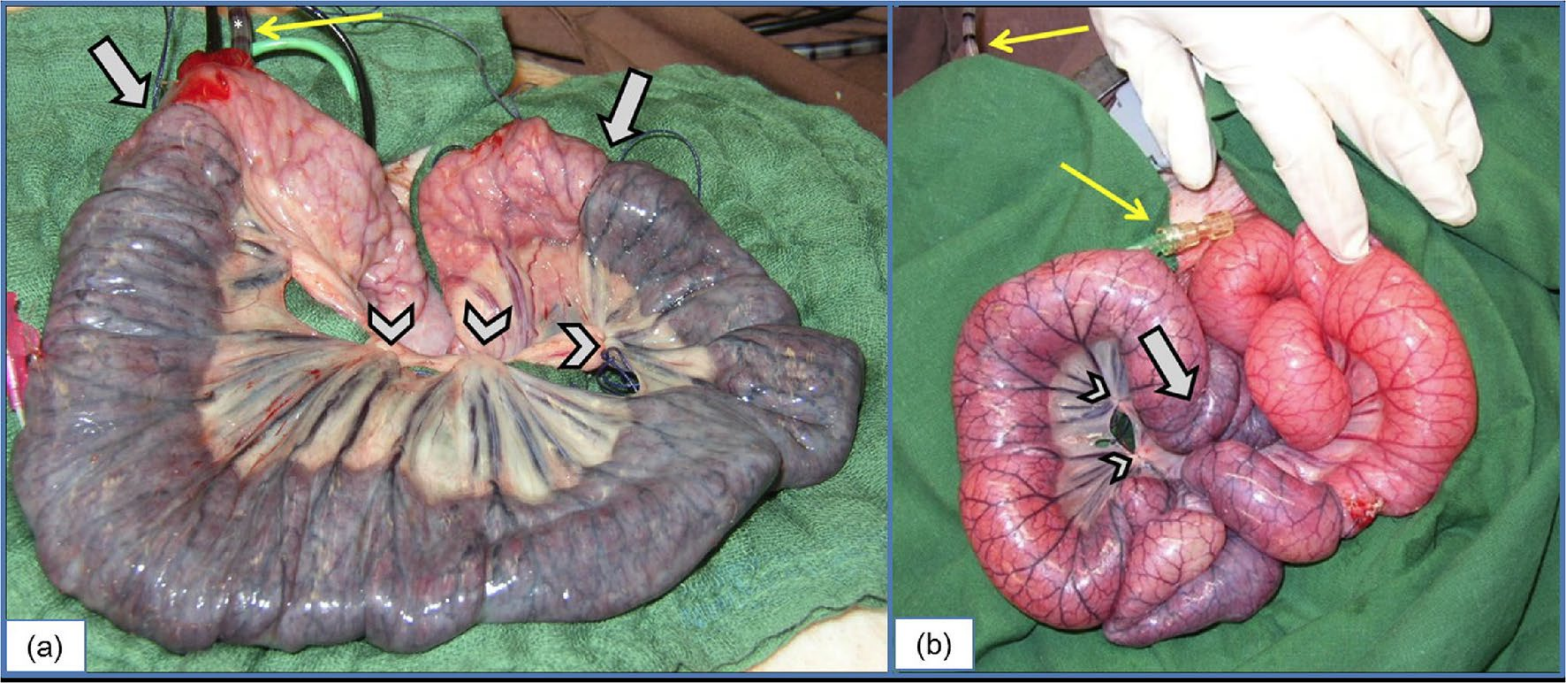
Clear macroscopic difference between treated and untreated bowel loops. (**a**) Appearance of the intestine after two hours of ischemia without treatment. Arrows point to the area where the organ was ligated. Arrowheads show mesenteric vessel ligations. Thin arrow shows catheters, emphasising the O_2_ supplier (*). (**b**) Treated small bowel after two hours of no-flow ischemia. Arrow shows one of the ligations around the organ to avoid collateral blood flow after ligation of mesenteric vessels (arrowheads). Thin arrows show the position of the oxygen-supplying catheter.

The isolated, non-perfused bowel segments of the sham group looked clearly ischemic (Fig. 2a). The bowel was flaccid on palpation and no peristalsis was observed. In contrast, the bowel segments perfused with oxygen appeared viable with normal colour, muscle tension and clearly visible peristaltic waves, albeit with some areas of hypoperfusion close to the ligatures and further from the centre of the segment (Fig. 2b).

### The protective effect of oxygen at microscopic level depends on luminal flow

Healthy tissue shows an overall median score of 0 (0–5). No differences were found when testing all control segments or twin central samples within each group (Supplementary Figure S2), so these were pooled for further analyses.

**Figure 3:**
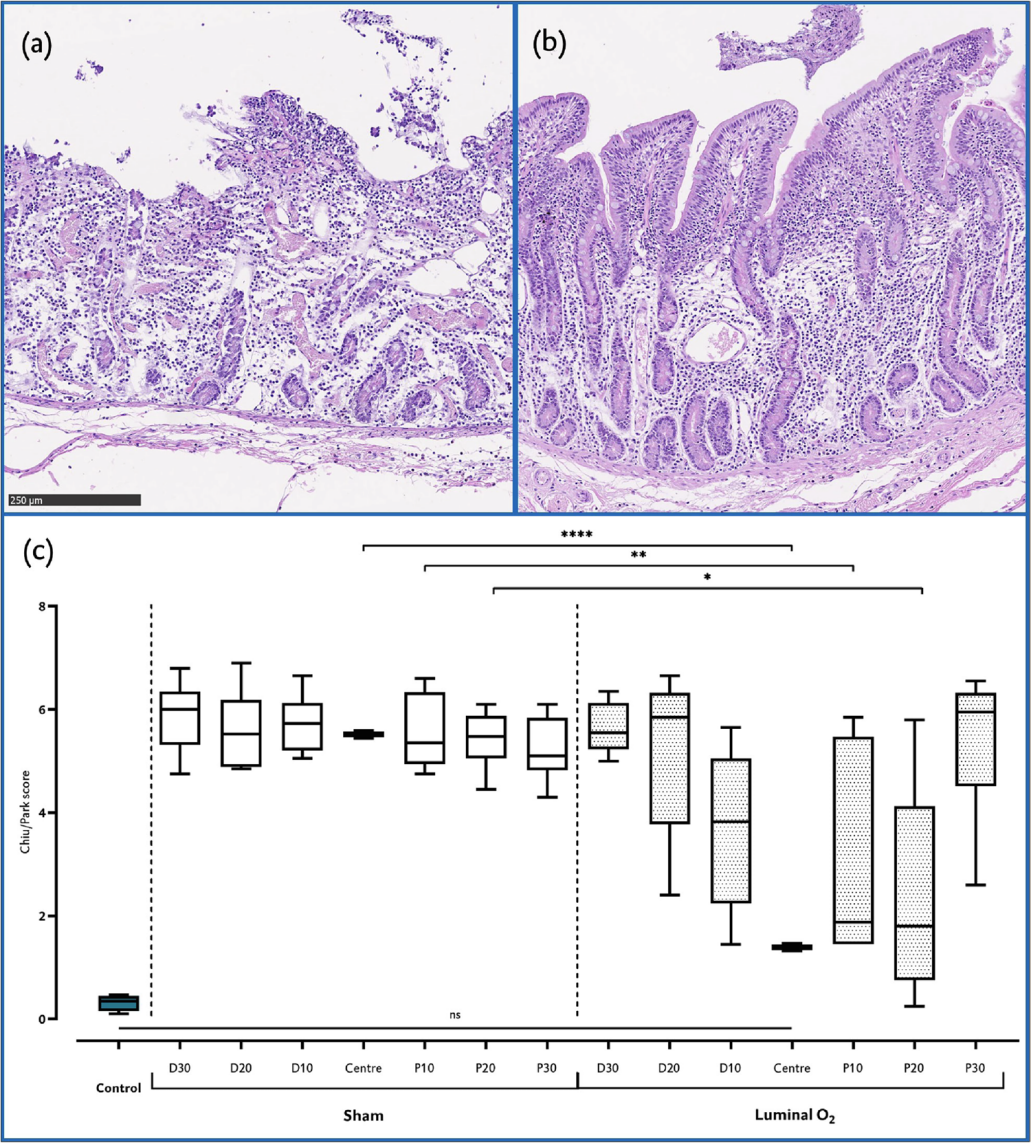
The mucosa is kept microscopically intact when in direct contact with luminal O_2_. Representative microscopic images (100 × light microscopy, haematoxylin–eosin staining) of the studied bowel segment. (**a**) Ischemic bowel after two hours of no-flow ischemia, centre. No epithelium, crypt destruction. Lamina propria shows hypocellularity, signs of oedema and increased fibrosis. (**b**) Central area of a bowel treated with luminal gaseous O_2_. A healthy mucosa with full epithelial lining, mild oedema of the lamina propria at level of the crypts was the most common finding. There is evidence of flaking in the lumen, although the origin of these cells is unclear. (**c**) Chiu/Park score of all sampled areas separated by distance and direction from the oxygen application catheter end: “control” are samples gathered outside of the ischemic area; “D” distal, “P” proximal, each number next to these letters represents the centimetres away from the “centre”, which is the area around the tip of the catheter. “Sham” are the untreated samples, “Luminal O_2_”, the treated ones. Each star represents a level of statistical significance: p < 0.0001 (****), p < 0.05 (*), not significant (“ns”).

In the sham group, the damage is homogeneous throughout the experimental segment (Chiu/Park score 5, 4–8, p < 0.0001, Fig. 3). There is a difference between the control samples and all untreated sections (p < 0.0001) which was also found on the treated samples in all distal (p ≤ 0.0001) and proximal 10 (p = 0.007) and 30 (p < 0.001) centimetres from the centre. However, the central samples of the treated loops show conserved architecture with few areas of focal damage. The lamina propria showed more conserved histology at villi than at crypt level, where more oedema could be seen., the tissue was found to be like healthy samples (1.39, 0–8, p = 0.911), also at the proximal 20 cm (2.31, 0–8, p = 0.187). Groups were stratified by pooling samples from equal distances from the catheter tip to check for influence of the direction of the gaseous flow. The centre of the treated subjects and the healthy samples again showed no difference (p = 0.604). Further away from the centre the protection of the oxygen ceased (p < 0.0001, Supplementary Figure S3).

The total amount of surface epithelium that was conserved was measured to account for areas without necrosis but with the lamina propria naked to the lumen^19^ (Supplementary Figure S4). No statistical differences between healthy and treated samples at the centre and up to 20 cm proximally and 10 cm distally were found. When samples were stratified, statistical significance appeared from 10 cm from the origin of oxygen administration onwards.

Another known reaction of the intestinal mucosa to ischemia are the villous length, reflecting the loss of villous tissue due to ischemia, and crypt depth, which increases as a response to ischemia because of the presence of intestinal pluripotent stem cells^19^. Healthy villous height was of 612.5 µm (529–692). Differences in these values were found both between control and sham and control and treated (p < 0.001) central samples. However, villi were significantly higher in the centre of the therapy group when compared to the untreated central samples (p = 0.0004, Supplementary Figure S5). This difference was not conserved further away from these areas. When pooling the samples by distance, the significance became larger (p = 0.0001). Regarding the crypt depth, the normal median value in healthy tissue was of 309.5 µm (253–365.3). There were differences between the control tissue and the untreated samples at the centre (p < 0.0001), but also with the treated samples (p = 0.0064). When comparing treated and untreated samples at the centre, a statistical difference was found, reflecting the presence of deeper crypts on the latter (162 versus 257 µm). These differences appeared up to 10 cm in each direction, also as the locations were stratified (Supplementary Figure S6).

Taken together, these results show a protective effect of gaseous luminal oxygen in ischemic bowel loops depending on the site and direction of application, with distances further from the centre being less protected, more so when the mucosa lies opposite to the direction of the flow.

### HIF 1-α expression is reduced and barrier function is preserved in luminal oxygenated small intestine

**Figure 4:**
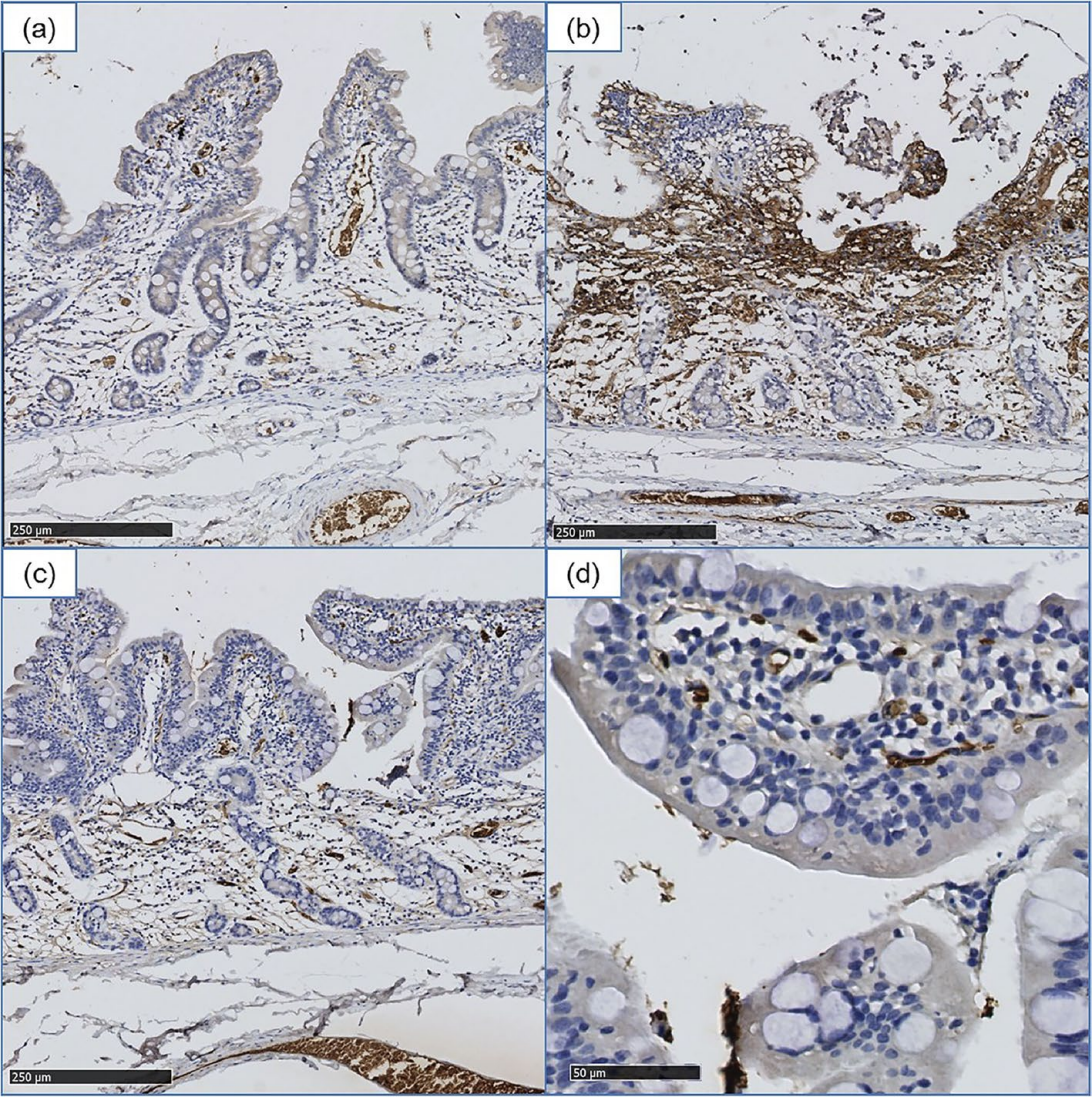
Less presence of HIF1-α in intestinal mucosa in direct contact with luminal oxygen according to immunohistochemistry compared to untreated bowels. 100 × magnification except where noted. (**a**) Control tissue. (**b**) Central area of the ischemic, untreated segments. Strong staining in the lamina propria and complete absence of the epithelial layer. (**c**) Treated bowel has a similar staining pattern as control samples, with areas of oedema and loss of enterocytes between the crypts and superficial epithelium. (**d**) Higher magnification of well-maintained villi tips of the treated group (**c**) with light cytoplasmic staining and strong positive staining of the erythrocytes present within the villi vessels (400x).

A staining for HIF1-α was performed to further specify whether there was a direct effect on oxygenation on the damage, or lack thereof, and to localise this damage when it occurred. Areas that were subjected to ischemia without the presence of oxygen showed more expression of this molecule. Oxygenated areas where the epithelium is still present and healthy samples show a mild cytoplasmic staining in enterocytes (Fig. 4).

To understand whether the maintained epithelial layer in treated samples kept barrier functional markers also intact, we stained for villin-1.

Villin-1 is present in the brush border of the enterocytes, giving an apical membranous staining in control samples. Staining is continuous and recedes when approaching the crypt epithelium. Treated samples show, when the epithelium is present, a thinner yet more intense staining (compact). The staining intensity decreases when reaching areas with no epithelium. This gradual pattern is not associated with the sudden loss of structure (Supplementary Figure S7).

Generally, these markers show a similar pattern of staining between control samples and areas where the epithelium is well maintained due to luminal oxygen treatment. Taken together, these results show the beneficial effect of luminal oxygen to maintain epithelial microstructure.

## Discussion

This study shows that intraluminal oxygen can preserve small bowel mucosal integrity and prevent ischemic damage during two hours of complete normothermic cessation of blood flow. We observed a protected small intestinal mucosa with this oxygen administration method, which is characterized by low-flow delivery. This delivery system may be clinically feasible and deserves further exploration in prevention of ischemia-related injury of the intestine. Current preservation methods for the intestinal graft do not consider the organ’s high mucosal energy requirements, so intraluminal preservation is not used in current ITx protocols. Because of its high oxygen consumption, luminal provision of oxygen could address these issues, particularly during the periods of warm ischemia.

Ischemia of the bowel which lasts more than an hour is associated with decreased patient survival when^4^. A faster transition to colder temperatures during organ procurement in multi organ donors can be addressed with the use of an ice-cold luminal preservation solution. In our study we show that luminal gaseous oxygenation can maintain the bowel’s integrity during prolonged periods of ischemia. This luminal delivery system could also improve the distribution of oxygen throughout the graft and delay the increase in temperature during implantation, thereby decreasing WIT. Studies with luminal preservation solutions applied during CIT have shown improvement of the state of the graft for transplantation^20–24^. To the extent of our knowledge, only one group experimented with oxygenated solutions such as perfluorocarbon. Because it has a high molecular weight (twice that of water), large volumes are needed^25–27^, uneven distribution of perfluorocarbon in the bowel with localized high-pressure zones is conceivable.

Luminal gaseous oxygen was tested in the past by the teams of Churchill and Minor, who successfully tested their methods in rats, showing improvement of function and reduction in oxidative damage after reperfusion^28,29^. The stronger presence of HIF1-α and villin point to an improvement in the preservation of function and lower oxidative damage in the treated segments in this current study.

The effects of reperfusion have not been addressed within this study. Since the model focuses on supplying a permanent flow of oxygen, IRI is expected not to be significant at the onset of reperfusion. Even so, the protective measures that come from the vascular flush are also not present, and, yet there is notable improvement in the macro- and microscopic evaluations of the organ. The presence of an epithelial layer and increased activity of the intestinal stem cells as reflected by the deeper crypts is an unequivocal sign of improvement and readiness for the reperfusion stage.

The preservation of a normal anaerobic healthy microbiota could be of concern when applying luminal oxygen^30^. Because of the state of the epithelial barrier with this treatment, and of the relatively short amount of time that is needed to treat the bowel during moments of normothermic ischemia (about an hour in most transplant centres), the influence of luminal oxygen on the composition of the microbiota is not expected to be significant.

Technical inconveniences must be addressed before any type of luminal preservation treatment can be applied in the clinics. The delivery method for any of these materials to the lumen must be quick and not interfere with the transplantation procedure. Our proposed technique relies mainly on luminal catheter placement. Most donors and ITx recipients already have catheters placed in their digestive tracts, so this method could be applied in situ in both. However, the length of the catheters should conform to intestinal segments treated, which in the case of adult intestinal transplantations can be quite long. The recipient’s stoma can also function as a readily available point of entry for an oxygen delivery method such as the one described herein in the case of post-surgical vascular complications. In our experiments, luminal oxygenation resulted in a macroscopically slightly inflated small bowel. The intraluminal pressure did not exceed 1 kPa during the experiments, but entrapment of oxygen in small bowel loops might in theory give rise to overpressure and complications. These concerns can be cleared with a dynamic method of application, seemingly necessary according to our results.

Intraluminal oxygenation may be of use for several other clinical conditions. The temporary mucosal ischemia in anastomotic areas and subsequent leakage is one of these challenges. In experimental and clinical studies, a consistent relationship between anastomotic ischemia and complications is reported^31–36^. Few models have tested the effects of intraluminal application of local oxygen distribution^22,25,37^. In clinical practice, blood flow remains at 10–40% of baseline^38^, whilst our model presents 100% cessation. Also, the ischemic area in this study is large in comparison with surgical anastomosis where the low flow area is restricted to a few centimetres at both sides^31^. The small amount of oxygen used in this experiment (5 ml/min) could be reduced even further for these cases, which would minimise those potential complications of gaseous oxygen application.

In summary, the results of this study demonstrate that in a pig model, intraluminal application of low flow of gaseous oxygen can keep the mucosa intact, preventing ischemic damage in an extreme model of jejunal ischemia using zero blood flow conditions. Ideally, oxygen should be administered in a dynamic way, since the distance from the application point and the flow direction seems to be of importance to reach the best possible results.

### Supplementary Information

Supplementary Figures.

Supplementary Tables.

## Data Availability

The datasets used and/or analysed during the current study available from the corresponding author on reasonable request.

## Abbreviations

CIT: Cold ischemia time
HIF1-α: Hypoxia-inducible factor 1-α
IRI: Ischemia–reperfusion injury
ITx: Intestinal transplant(ation)
PEEP: Positive end-expiratory pressure
WIT: Warm ischemia time
