# Supplementary material for: Intraluminal oxygen can keep small bowel mucosa intact in a segmental ischemia model
Source: Sci Rep. 2024 Jun 14;14:13732. doi: 10.1038/s41598-024-64660-x (PMC11178904; doi:10.1038/s41598-024-64660-x)
Supplement: Supplementary file 1 — Supplementary Figures. [file 41598_2024_64660_MOESM1_ESM.pdf]

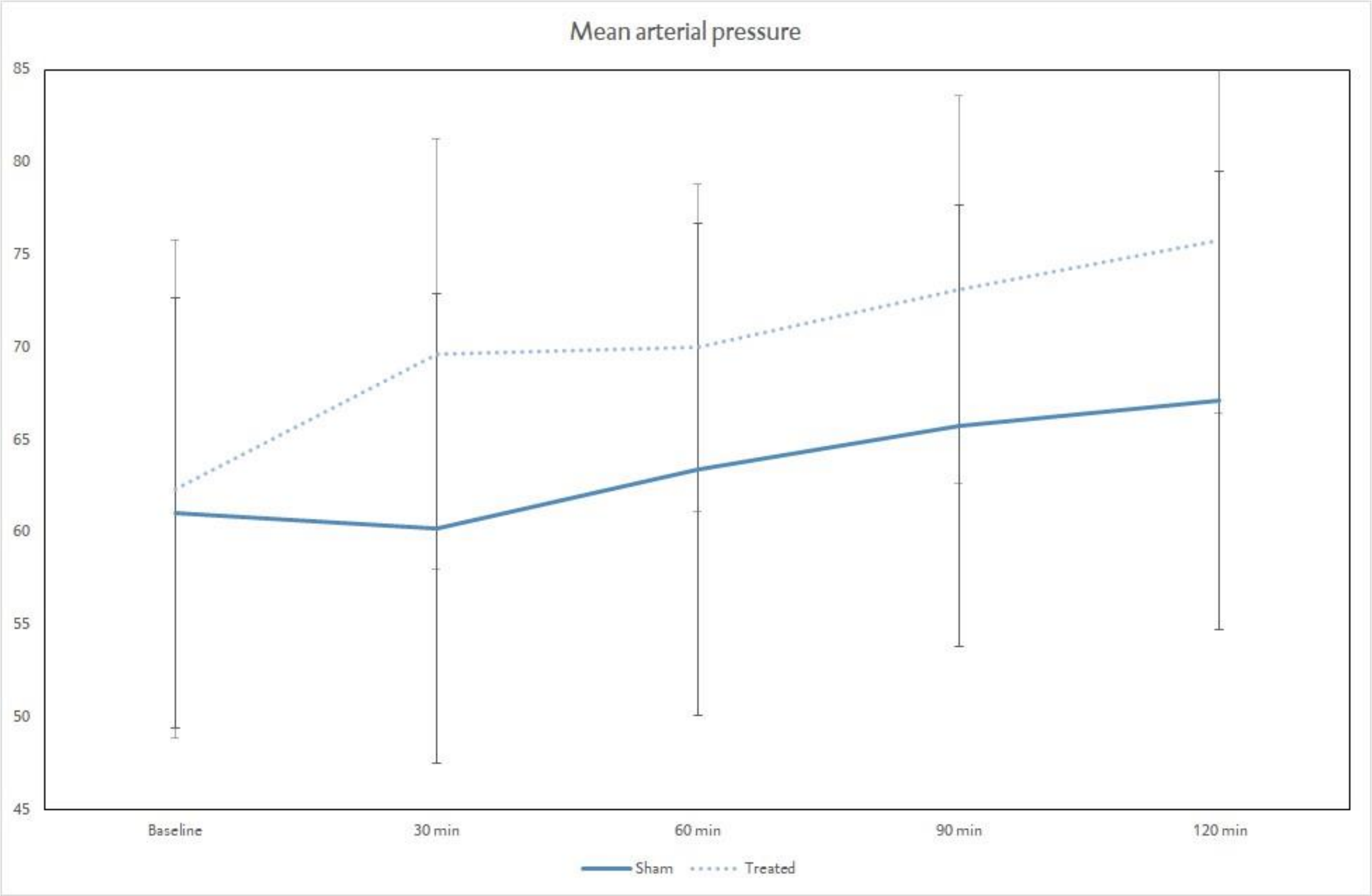

Figure S1. Mean arterial pressure measurements in both sham and treated pigs show no statistical differences between groups during the two-hour procedure, but a slight increase over time (p=0.006).

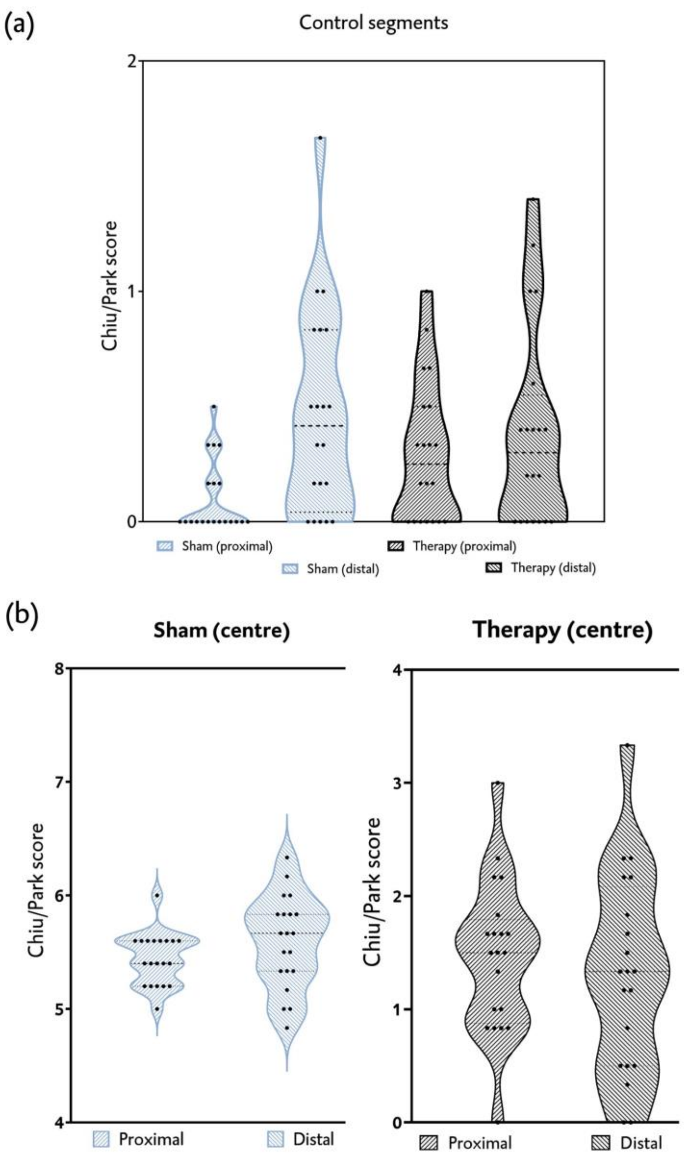

Figure S2. Park/Chiu scores of biological duplicates: **(a)** control tissue, outside of the studied loop, shows healthy scores and no statistical difference. **(b)** Central tissue samples in two different areas also show no differences within groups. **Left**: central areas of sham group mucosa show high scores. **Right**: also no difference within the treated group, showing healthy scores.

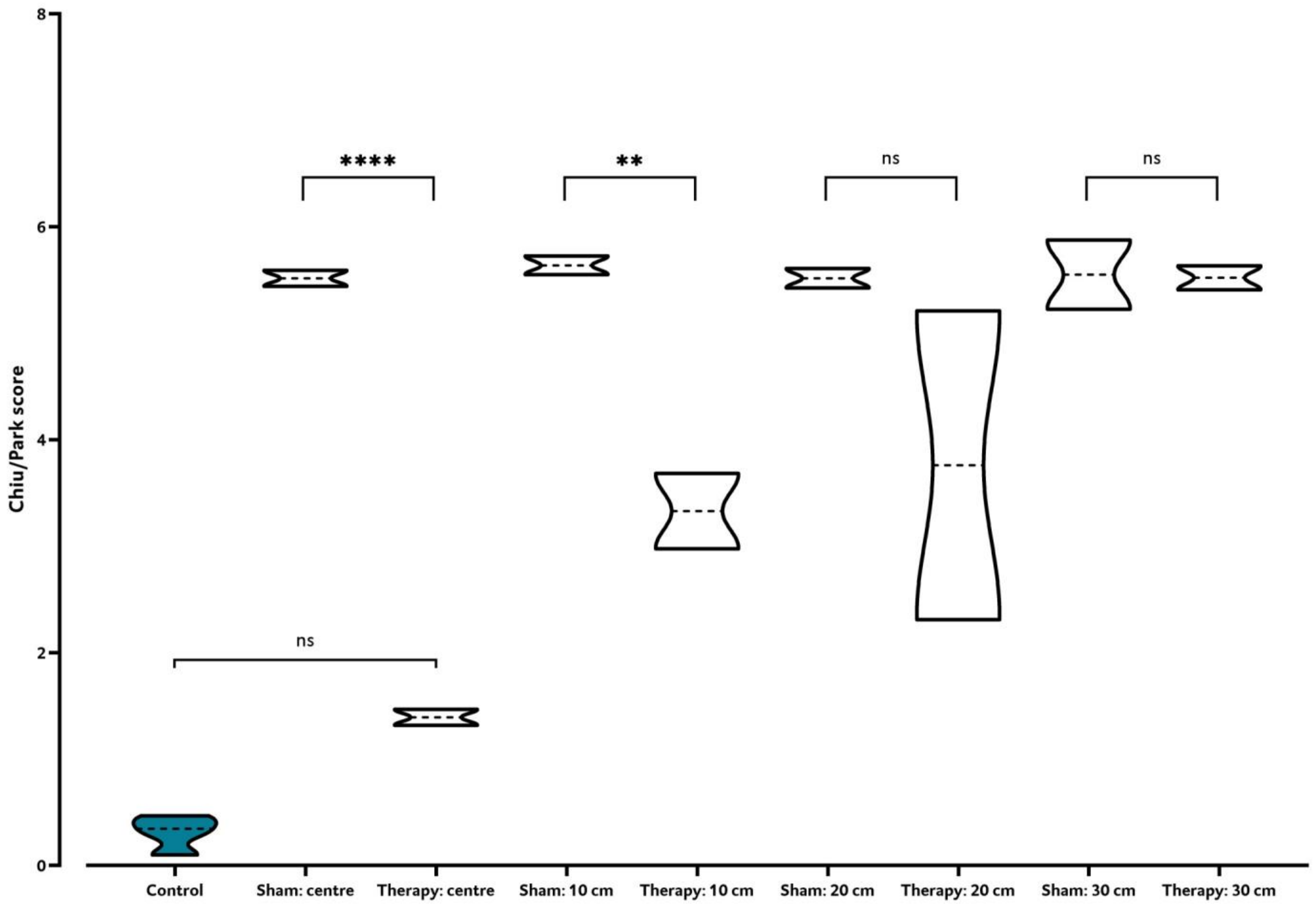

Figure S3. Pooled areas according to the distance from oxygen supply show a beneficial effect of proximity to the catheter tip. Control samples were taken outside of the ischemic areas. “ns” non-significant statistical differences; p<0.0001 (****); p<0.001 (**).

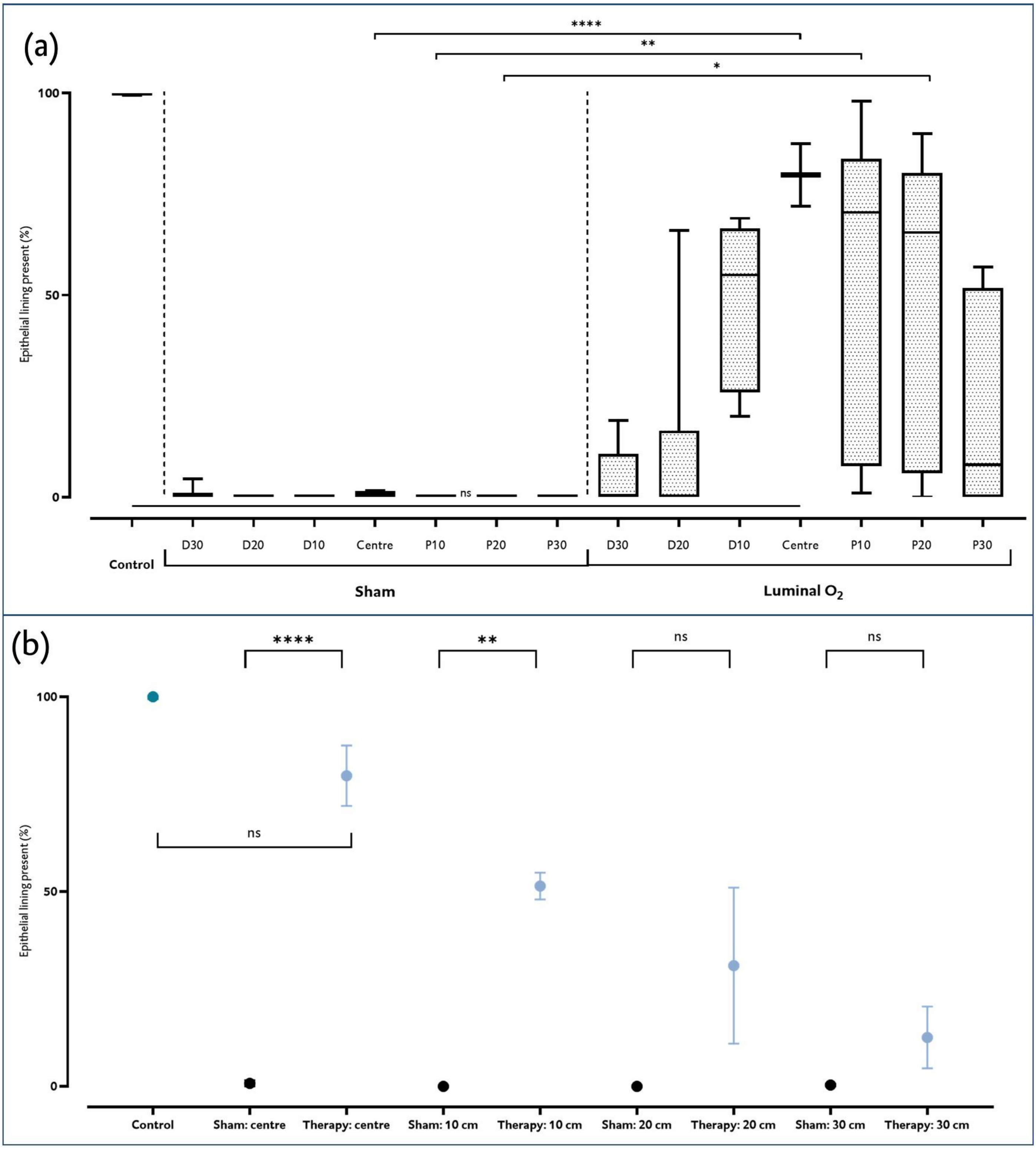

Figure S4. Morphological analyses of histological samples: presence of intact epithelial lining (percentage of the whole sample) is higher in treated samples. **(a)** Individual localisations of both treated and non-treated bowel segments and score of control samples. **(b)** Scores when stratifying localisations depending on the distance from oxygen application but not the direction. “Control” are samples gathered outside of the ischemic area; “D” distal, “P” proximal, each number next to these letters represents the centimetres away from the “centre”, which is the area around the tip of the catheter. “Sham” are the untreated samples, “Luminal O_2”, the treated ones. Each star represents a level of statistical significance: p<0.0001 (****), p<0.05 (*), not significant (“ns”).

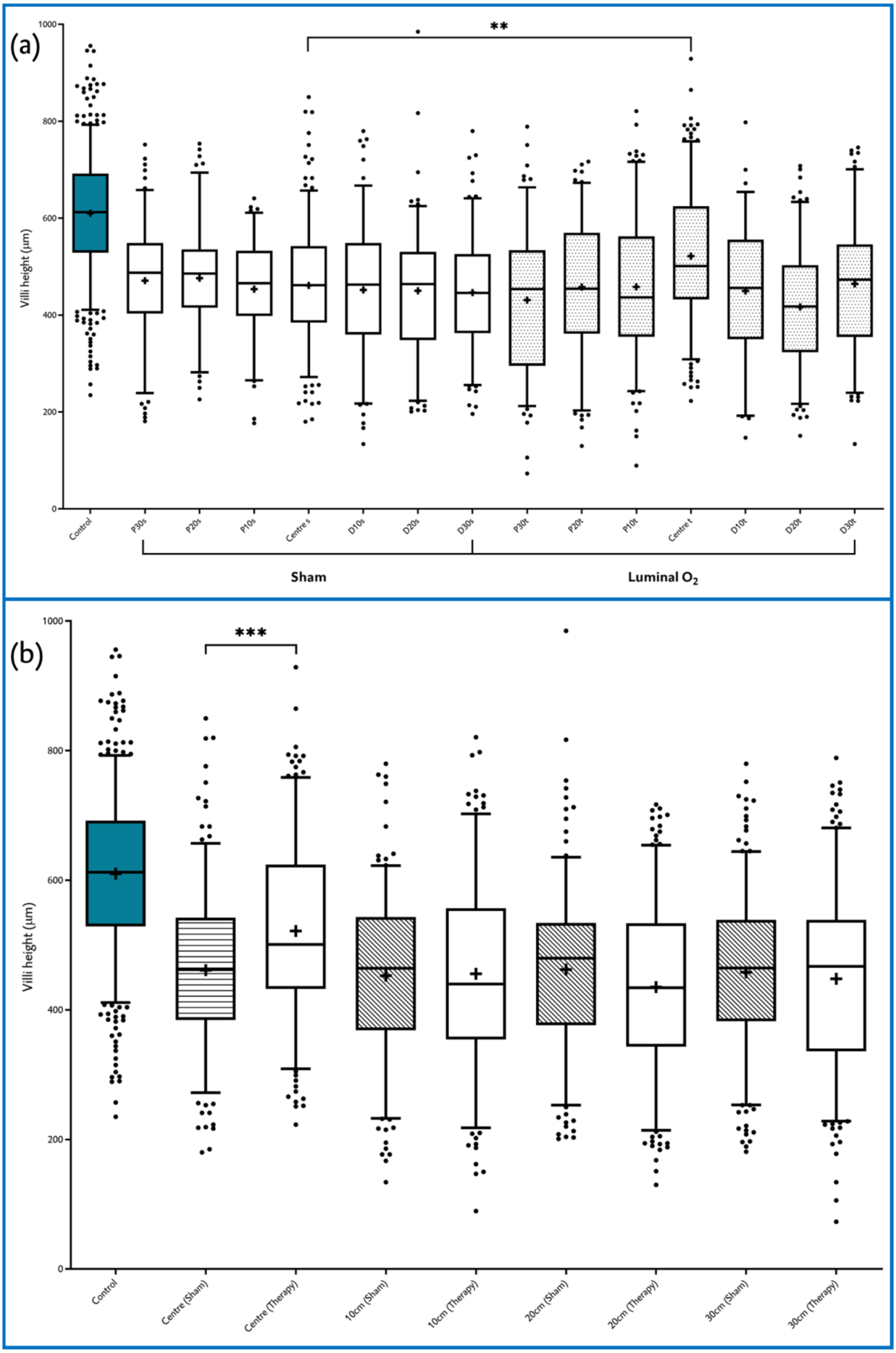

Figure S5. Morphological analyses of histological samples: villi height is larger in treated samples at the centre when compared to untreated samples **(a)** Individual localisations of both treated and non-treated bowel segments and score of control samples. **(b)** Scores when stratifying localisations depending on the distance from oxygen application but not the direction. "Control" are samples gathered outside of the ischemic area; "D" distal, "P" proximal, each number next to these letters represents the centimetres away from the "centre", which is the area around the tip of the catheter. "Sham" are the untreated samples, "Luminal O_2", the treated ones. Each star represents a level of statistical significance: $p<0.001$ (***), $p<0.05$ (**).

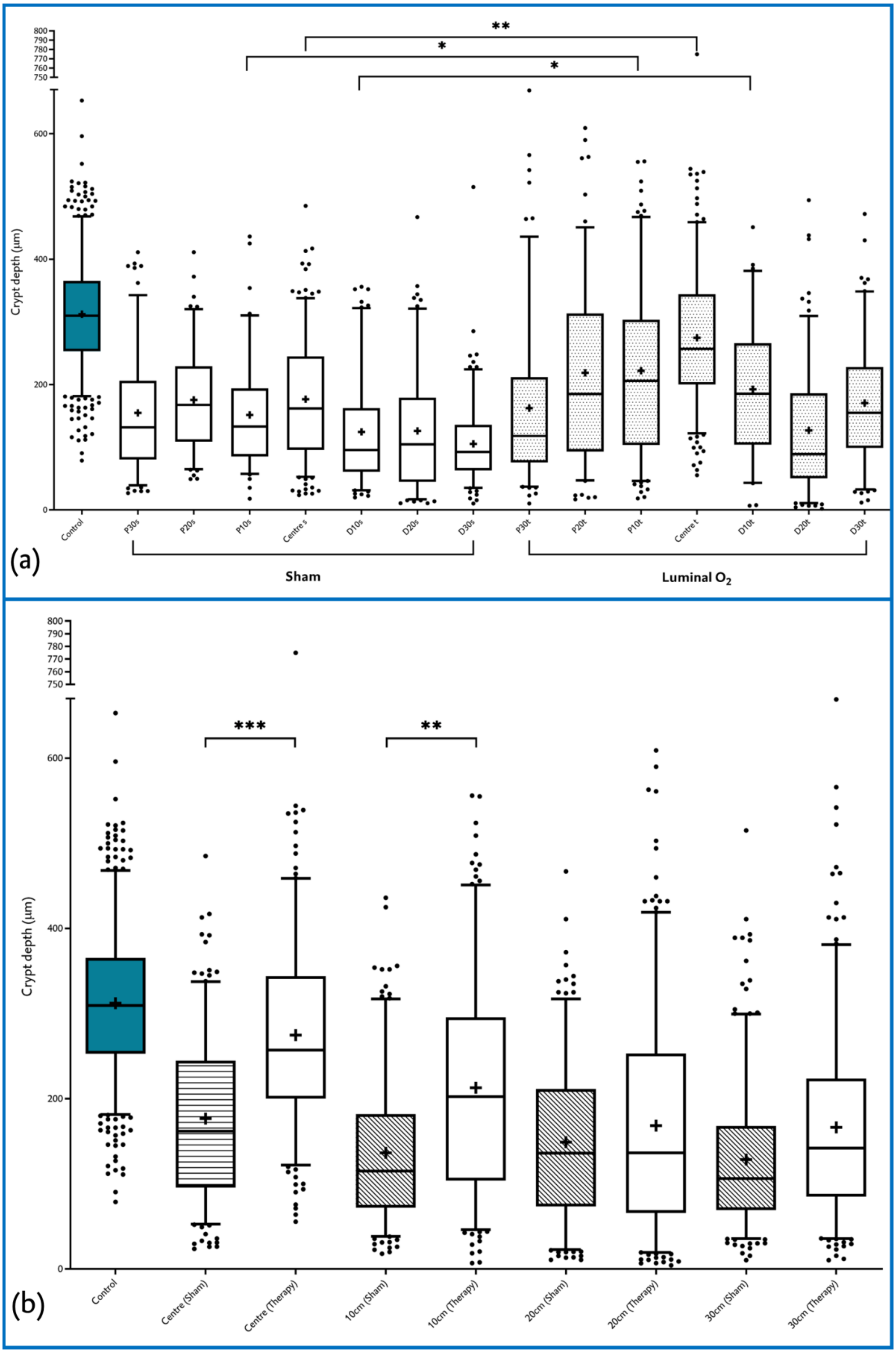

Figure S6. Morphological analyses of histological samples: crypts are deeper in treated samples at the centre and up to ten centimetres away when compared to untreated samples **(a)** Individual localisations of both treated and non-treated bowel segments and score of control samples. **(b)** Scores when stratifying localisations depending on the distance from oxygen application but not the direction. "Control" are samples gathered outside of the ischemic area; "D" distal, "P" proximal, each number next to these letters represents the centimetres away from the "centre", which is the area around the tip of the catheter.

“Sham” are the untreated samples, “Luminal O_2”, the treated ones. Each star represents a level of statistical significance: $p<0.0001$ (***), $p<0.001$ (**), $p<0.05$ (*).

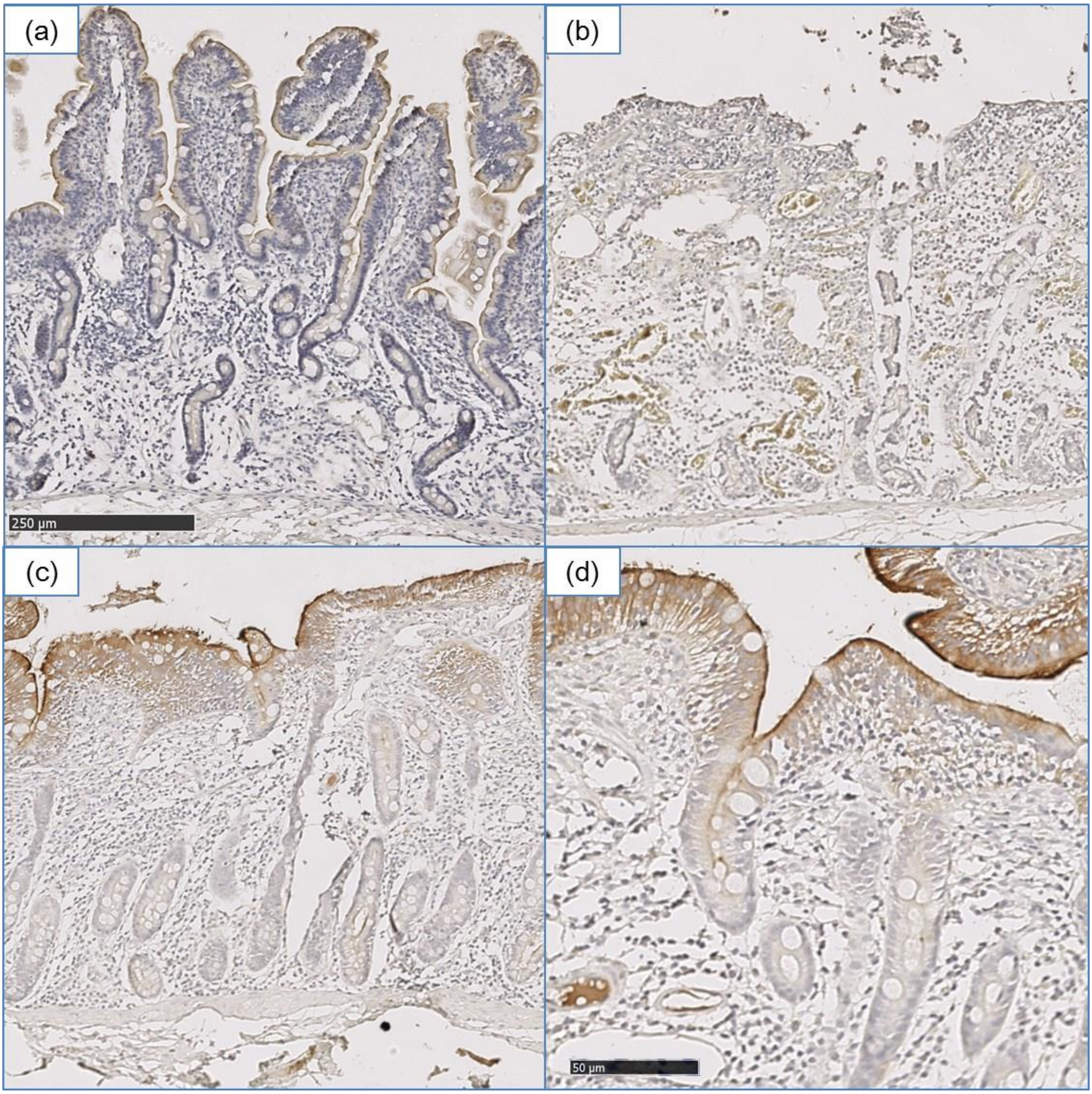

Figure S7. Immunohistochemical staining for villin-1 showing similarities between control and treated samples. **(a)** Low magnification (100x) of a control sample showing normal villi structure and strong apical staining of enterocytes. **(b)** Sample of ischemic, non-treated mucosa without epithelium left (100x). **(c)** Staining pattern from the treated central areas of the ischemic bowel segments (100x) and **(d)** higher magnification of the same sample.

(a)

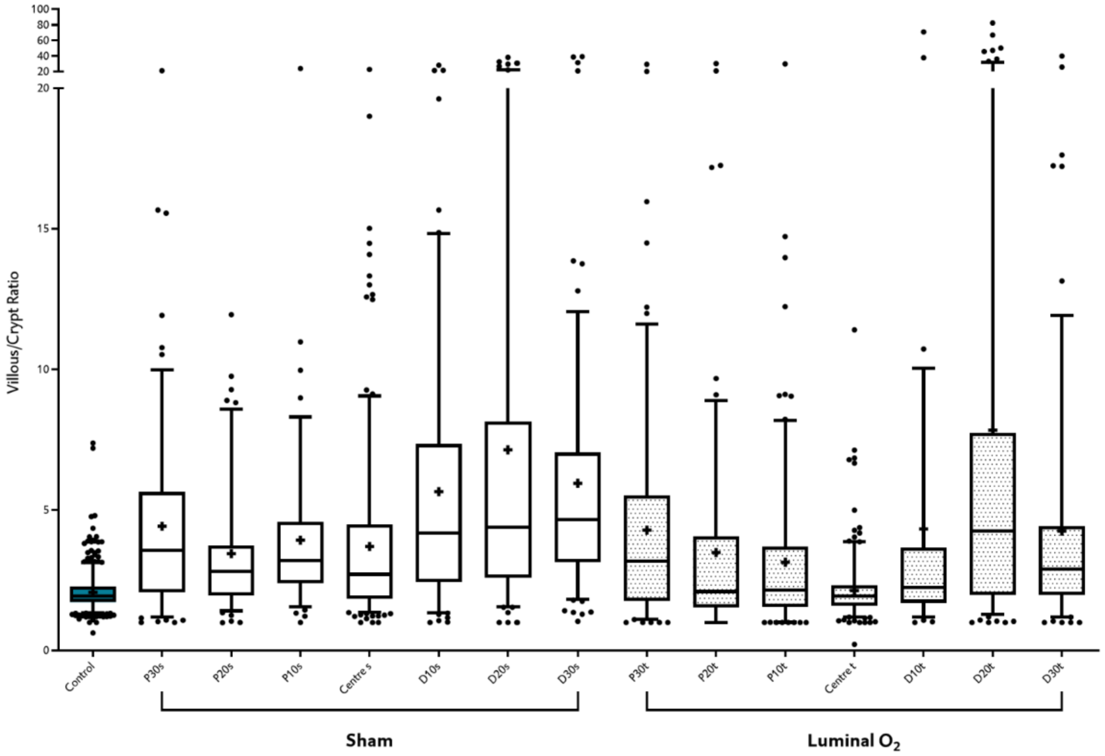

(b)

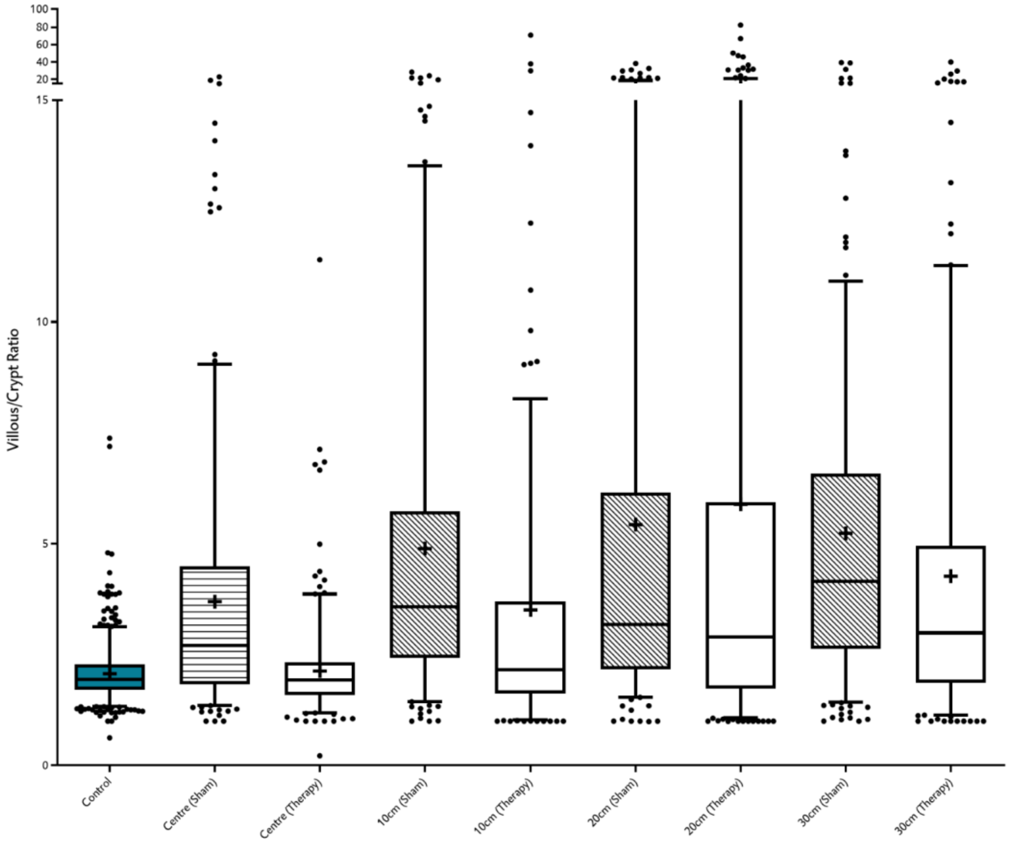

Figure S8. Villous to crypt ratio ("VCR") shows differences between treated and untreated samples. Morphological analyses of histological samples: villi to crypt ratio (VCR). **(a)** Individual localisations of both treated and non-treated bowel segments and score of control samples. **(b)** Scores when stratifying localisations depending on the distance from oxygen application but not the direction.

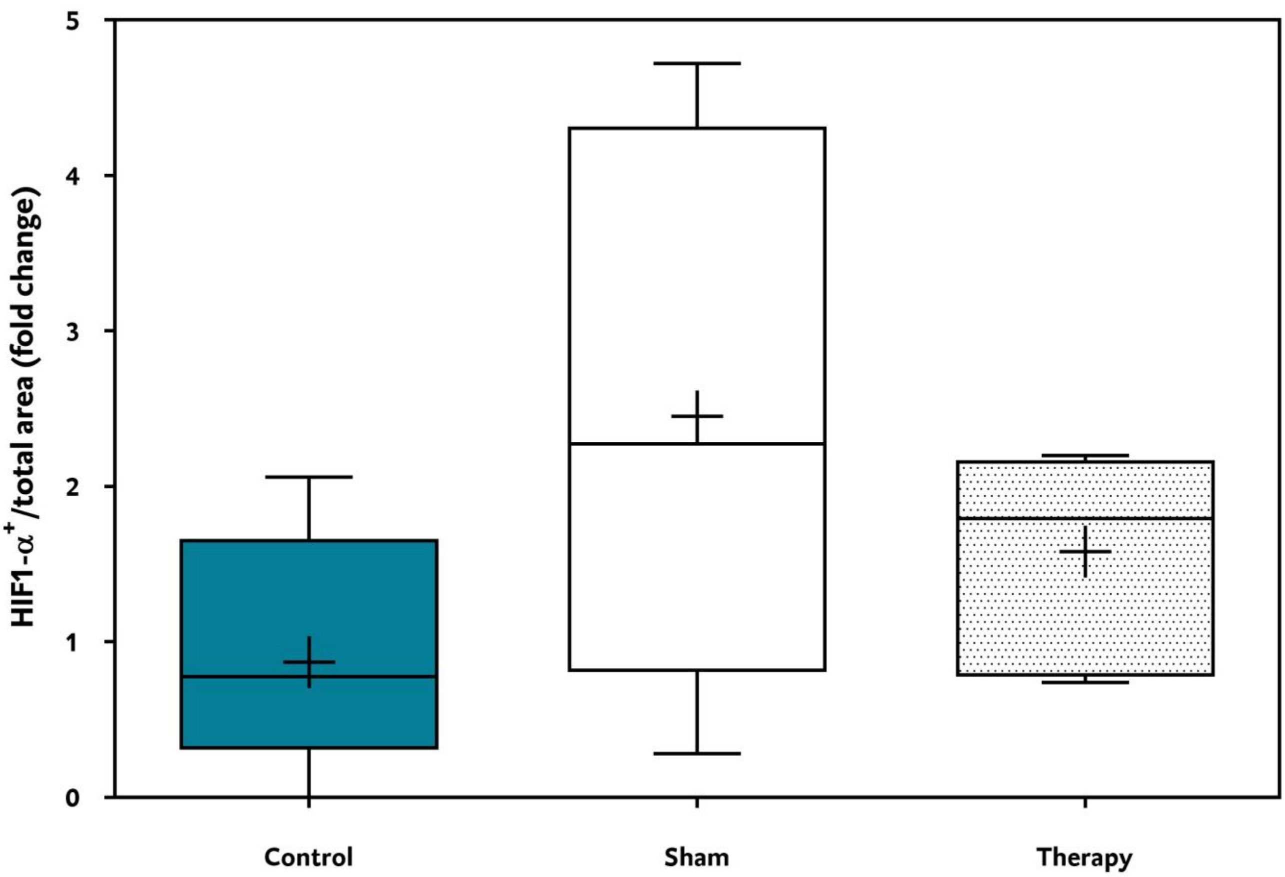

Figure S9. HIF1-α is more present in central samples that were not treated with luminal oxygen. Relation between area (mm2) stained with DAB and the total area analysed from segments of each of the central and untreated areas of both study groups. n=30 per group, where each subject (n=6 per group) was evaluated in 5 different areas. Untreated areas from both groups have been merged into one group (n=60). Quantitative method to measure the presence of DAB within the mucosa shows that treated samples ("Therapy") have less stained area than untreated bowels ("Sham"). Values are similar to that of healthy samples ("Control").

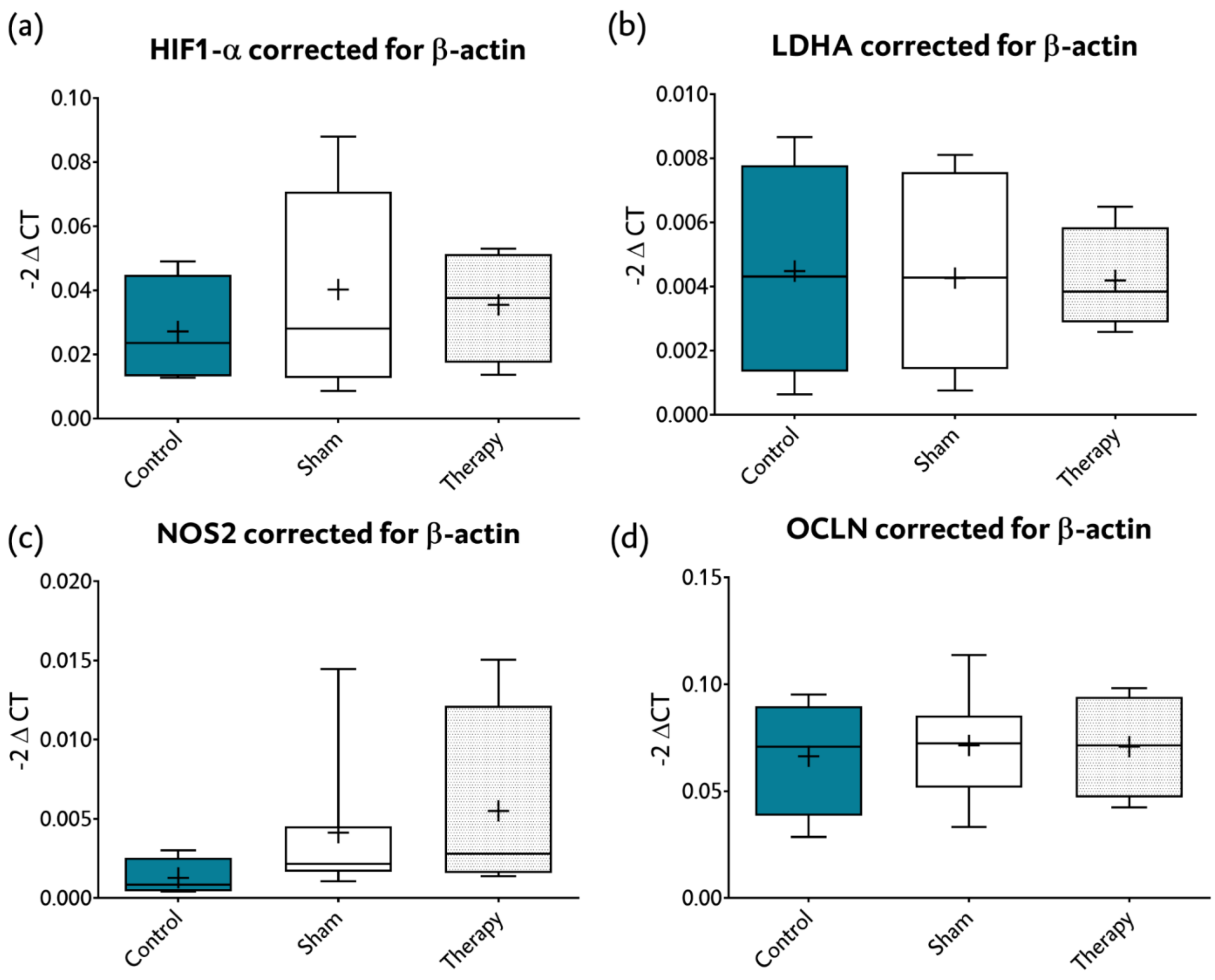

Figure S10. Gene expression profiles for three ischemia-related genes and one barrier function-related gene (occludin). These results are expressed relative to β-actin. "Control" are samples outside of the studied areas, non-ischemic; "Sham" are samples from the central area of the non-treated ischemic bowel segments; "Therapy" samples were taken from the centre of the oxygenated ischemic bowel segment. **(a)** hypoxia-induced factor 1-α; **(b)** lactate-dehydrogenase A; **(c)** nitric-oxide synthase 2; **(d)** occludin.
