# Supplementary material for: Intraluminal oxygen can keep small bowel mucosa intact in a segmental ischemia model
Source: Sci Rep. 2024 Jun 14;14:13732. doi: 10.1038/s41598-024-64660-x (PMC11178904; doi:10.1038/s41598-024-64660-x)
Supplement: Supplementary file 2 — Supplementary Tables. [file 41598_2024_64660_MOESM2_ESM.docx]

# Supplementary Tables

## ***Table S1.***

| ***Grade*** | ***Description*** |
| --- | --- |
| 0 | Normal mucosa |
| 1 | Subepithelial space at villous tips |
| 2 | Epithelial lifting at villous tips |
| 3 | Epithelial lifting alongside villous sides |
| 4 | Denudation of villi (necrosis) |
| 5 | Loss of villous tissue |
| 6 | Crypt layer infarction |
| 7 | Transmucosal infarction |
| 8 | Transmural infarction |

## ***Table S2.***

| **Group** | ***Sham (n = 6)*** | | | | | | | ***Therapy (n = 6)*** | | | | | | |
| --- | --- | --- | --- | --- | --- | --- | --- | --- | --- | --- | --- | --- | --- | --- |
| Time point | t = 0 | Min | Max | t = 120 | Min | Max | T test | t = 0 | Min | Max | t = 120 | Min | Max | T test |
| HR (beats/min) | 64,50 | 50 | 75 | 73,50 | 46 | 103 | 0,24 | 56,00 | 50 | 79 | 60,50 | 52 | 90 | 0,18 |
| MAP (mmHg) | 57,50 | 49 | 80 | 67,50 | 52 | 86 | 0,19 | 57,33 | 51 | 90 | 73,67 | 67 | 94 | 0.01* |
| Art BE | 3,58 | 3 | 6 | 4,53 | 3 | 7 | 0,08 | 4,77 | 2 | 6 | 4,44 | 2 | 7 | 0,59 |
| Art Ca^2+^ (mg/dl) | 1,37 | 1 | 1 | 1,41 | 1 | 1 | 0.02* | 1,37 | 1 | 1 | 1,42 | 1 | 2 | 0,06 |
| Art Cl^-^ (mmol/dl) | 104 | 99 | 106 | 103 | 101 | 106 | 0,77 | 103 | 101 | 107 | 103 | 102 | 106 | 0,53 |
| Art Glu (mg/dl) | 92,00 | 78 | 151 | 113,50 | 82 | 137 | 0,13 | 104,50 | 70 | 135 | 112,50 | 97 | 126 | 0,45 |
| Art HCO_3_ (mmol/dl) | 27,40 | 27 | 29 | 28,07 | 27 | 30 | 0.04* | 28,64 | 26 | 30 | 28,24 | 26 | 29 | 0,85 |
| Art Hct | 26,15 | 20 | 27 | 25,45 | 21 | 28 | 0,84 | 24,25 | 22 | 27 | 23,85 | 22 | 29 | 0,71 |
| Art K^+^ (mmol/dl) | 4,10 | 3 | 4 | 4,45 | 4 | 5 | 0.02* | 3,80 | 4 | 4 | 4,30 | 4 | 5 | 0.03* |
| Art Na^+^ (mmol/dl) | 140,00 | 136 | 145 | 139,00 | 136 | 140 | 0,19 | 140,00 | 139 | 141 | 139,00 | 138 | 140 | 0,18 |
| Art pCO_2_ (mmHg) | 39,85 | 36 | 44 | 39,75 | 36 | 49 | 0,34 | 40,75 | 37 | 46 | 39,55 | 36 | 48 | 0,67 |
| Art pH | 7,45 | 7 | 8 | 7,45 | 7 | 8 | 0,95 | 7,46 | 7 | 7 | 7,45 | 7 | 8 | 0,53 |
| Art pO_2_ (mmHg) | 206,95 | 141 | 253 | 225,90 | 152 | 264 | 0,82 | 210,70 | 179 | 248 | 226,55 | 171 | 261 | 0,07 |
| Art SO_2_ (mmHg) | 100 | 100 | 100 | 100 | 100 | 100 |  | 100 | 100 | 100 | 100 | 100 | 100 |  |
| Art tHb (g/dl) | 8,40 | 6 | 9 | 8,20 | 7 | 9 | 0,84 | 7,75 | 7 | 9 | 7,65 | 7 | 9 | 0,65 |
| Art Lactate | 1,30 | 1 | 4 | 0,85 | 1 | 2 | 0,11 | 1,10 | 1 | 2 | 0,90 | 1 | 1 | 0,06 |
